# CXCR2 is essential for cerebral endothelial activation and leukocyte recruitment during neuroinflammation

**DOI:** 10.1186/s12974-015-0316-6

**Published:** 2015-05-21

**Authors:** Fengjiao Wu, Yawei Zhao, Tian Jiao, Dongyan Shi, Xingxing Zhu, Mingshun Zhang, Meiqing Shi, Hong Zhou

**Affiliations:** Department of Immunology, Nanjing Medical University, 140 Hanzhong Road, Nanjing, JS 210029 China; Division of Immunology, Virginia-Maryland College of Veterinary Medicine, University of Maryland, College Park, Maryland, MD 20742 USA

**Keywords:** CNS inflammation, CXCL1, CXCR2, Astrocyte, Endothelial activation, Leukocyte recruitment, Intravital microscopy

## Abstract

**Background:**

Chemokines and chemokine receptors cooperate to promote immune cell recruitment to the central nervous system (CNS). In this study, we investigated the roles of CXCR2 and CXCL1 in leukocyte recruitment to the CNS using a murine model of neuroinflammation.

**Methods:**

Wild-type (WT), CXCL1^−/−^, and CXCR2^−/−^ mice each received an intracerebroventricular (i.c.v.) injection of lipopolysaccharide (LPS). Esterase staining and intravital microscopy were performed to examine neutrophil recruitment to the brain. To assess endothelial activation in these mice, the expression of adhesion molecules was measured via quantitative real-time polymerase chain reaction (PCR) and Western blotting. To identify the cellular source of functional CXCR2, chimeric mice were generated by transferring bone marrow cells between the WT and CXCR2^−/−^ mice.

**Results:**

Expression levels of the chemokines CXCL1, CXCL2, and CXCL5 were significantly increased in the brain following the i.c.v. injection of LPS. CXCR2 or CXCL1 deficiency blocked neutrophil infiltration and leukocyte recruitment in the cerebral microvessels. In the CXCR2^−/−^ and CXCL1^−/−^ mice, the cerebral endothelial expression of adhesion molecules such as P-selectin and VCAM-1 was dramatically reduced. Furthermore, the bone marrow transfer experiments demonstrated that CXCR2 expression on CNS-residing cells is essential for cerebral endothelial activation and leukocyte recruitment. Compared with microglia, cultured astrocytes secreted a much higher level of CXCL1 in vitro. Astrocyte culture conditioned medium significantly increased the expression of VCAM-1 and ICAM-1 in cerebral endothelial cells in a CXCR2-dependent manner. Additionally, CXCR2 messenger RNA (mRNA) expression in cerebral endothelial cells but not in microglia or astrocytes was increased following tumor necrosis factor-α (TNF-α) stimulation. The intravenous injection of the CXCR2 antagonist SB225002 significantly inhibited endothelial activation and leukocyte recruitment to cerebral microvessels.

**Conclusions:**

CXCL1 secreted by astrocytes and endothelial CXCR2 play essential roles in cerebral endothelial activation and subsequent leukocyte recruitment during neuroinflammation.

## Background

Immune cell recruitment is a key event in the development of many types of central nervous system (CNS) inflammatory diseases, such as bacterial meningitis [1], stroke [2], and multiple sclerosis [3]. Following the detection of pathogen-derived components or danger signals, leukocyte recruitment to the brain via chemotaxis [4, 5] occurs via a cascade-like process that involves the expression of endothelial cell- and leukocyte-expressed adhesion molecules such as selectins and integrins [6–8]. During the early stage of CNS inflammation, the interactions between chemokines and their receptors also exert a profound effect by attracting immune cells to migrate across the blood–brain barrier (BBB) [9, 10].

CXCR2 is a G protein-coupled receptor that is activated by CXC chemokines, including murine CXCL1, CXCL2, and CXCL5 [11, 12]. Interactions between CXCR2 and its ligands play an essential role in mediating neutrophil migration to sites of inflammation. Although extensive studies have focused on the role of CXCR2 in inflammatory responses in different organs, the involvement of individual chemokines in different types of inflammatory responses remains contentious. For example, CXCL1 is essential for the host pulmonary defense to klebsiella infection [13] and mediates neutrophil recruitment during the progression of experimental Lyme arthritis [14]. However, even in the presence of high levels of CXCL1 expression, the interaction between CXCL2 and CXCR2 is still essential for neutrophil migration in response to specific antigen challenge [15]. Additionally, CXCL2 plays a more important role than CXCL1 in a viral antigen-induced delayed-type hypersensitivity response [16]. Furthermore, CXCR2 is widely expressed on neutrophils [17], lymphocytes [18], and other types of non-hematopoietic cells, including epithelial [19] and endothelial cells [20, 21]. Most studies have focused on the functions of CXCR2 expressed on hematopoietic cells, such as monocytes [22, 23] and neutrophils [24–27]. However, recent studies have revealed a critical role of CXCR2 expressed on non-hematopoietic cells during inflammatory responses. In a murine model of acute kidney infection, CXCR2 on non-bone marrow-derived cells influenced the neutrophil response [19]. Additionally, CXCR2 expression on resident cells is essential for the migration of mast cell progenitors in the lung of antigen-challenged mice [28]. However, the roles of CXCR2 and its ligands in CNS inflammation remain to be addressed.

In this study, we performed intravital microscopy to examine the role of CXCR2 in leukocyte recruitment during neuroinflammation. We observed reduced neutrophil infiltration and attenuated leukocyte–endothelial cell interactions in CXCR2^−/−^ and CXCL1^−/−^ mice following the intracerebroventricular (i.c.v.) injection of lipopolysaccharide (LPS). Moreover, CXCR2 or CXCL1 deficiency impaired endothelial activation by attenuating the expression of adhesion molecules. Using chimeric mice expressing CXCR2 on either hematopoietic cells or radiation-resistant non-hematopoietic cells, we showed that CXCR2 expression on radiation-resistant cells in the CNS is essential for endothelial activation and subsequent leukocyte recruitment during neuroinflammatory responses. Furthermore, a high level of CXCL1 was detected in primary astrocyte culture and culture conditioned medium significantly increased the expression of VCAM-1 and ICAM-1 on cerebral endothelial cells. Taken together, our findings revealed a previously unrecognized role of CXCR2 expressed on cerebral endothelial cells in the regulation of endothelial activation and immune cell recruitment across the BBB during CNS inflammation.

## Methods

### Animals

C57BL/6J mice (8 to 10 weeks old, 22 to 25 g) used as wild-type controls were purchased from the Model Animal Research Center of Nanjing University. CXCR2^−/−^ mice (on the C57BL/6J background) were purchased from the Jackson Laboratory (Bar Harbor, ME, USA). All mice were maintained under environmentally controlled conditions (ambient temperature, 22 ± 2 °C; humidity 40 %) in a pathogen-free facility with a 12-h light/dark cycle and had access to water and food ad libitum. All experimental procedures were performed in strict accordance with the Institutional Animal Care and Use Committee of Nanjing Medical University.

### TALEN-mediated generation of CXCL1 knockout mice

To target the CXCL1 gene in the mouse genome, we designed and synthesized highly active TALENs specific to the CXCL1 gene. The TALEN target sequence for CXCL1 was GATCCCAGCCACCCGC. TALEN messenger RNAs (mRNAs) were diluted in RNase-free phosphate-buffered saline (PBS) and then injected into the cytoplasm of mouse pronuclear stage embryos to produce mutant founders (F0). Heterozygous F1 offspring were interbred to produce homozygous F2 animals. To functionally validate the TALEN-induced mutations, we intracerebroventricularly injected LPS into these mice and measured the level of CXCL1 in the brain. No expression of CXCL1 was detected in the CXCL1 mutant founder. The CXCL1^−/−^ mice were viable and fertile and did not exhibit any gross abnormalities.

### Intracerebroventricular injection of LPS

Intracerebroventricular injections into the mice were performed as previously described [29]. Briefly, the mice were anesthetized via intraperitoneal (i.p.) injection with a mixture of 200 mg/kg ketamine and 10 mg/kg xylazine. Then, 2 μg of LPS (dissolved in sterile saline at a concentration of 1 μg/μl; *Escherichia coli* serotype 0111:B4 strain; Invivogen, Carlsbad, CA, USA) was injected into the left ventricles using a microsyringe over a 5-min period. Sham mice received isovolumetric sterile saline injection. After LPS injection, the mice were maintained under anesthesia at 36 ± 1 °C on a thermostatic heating system (Harvard Apparatus, MA, USA) for 4 h before intravital microscopy was performed.

### ELISA for chemokines

The mice were anesthetized after LPS injection and subsequently perfused through the heart with 20 ml of cold PBS over a period of 5 min to remove protein from the blood circulation. Mouse brains were homogenized in 1 ml of cold PBS and centrifuged at 12,000 rpm for 5 min at 4 °C. The CXCL1, CXCL2, and CXCL5 concentrations in the supernatant were measured using commercial enzyme-linked immunosorbent assay (ELISA) kits (R&D systems, Minneapolis, MN, USA) according to the manufacturer’s instructions. The detection limit was 15.6 pg/ml for all assays.

### Flow cytometry

Flow cytometric analysis of single-cell suspensions prepared from peripheral blood or spleens of wild-type or CXCL1^−/−^ mice was performed on a Beckman CytoFlex (Beckman Coulter, Suzhou, China). Antibody clones used for staining were specific for Gr-1 (RB6-8C5, eBioscience, San Diego, USA), CD45 (30-F130, eBioscience), and CXCR2 (TG11, Biolegend, San Diego, CA, USA).

### Immunohistochemistry

After anesthetization, the mice were transcardially perfused with ice-cold 4 % formalin. Then, the brains were removed and fixed in 4 % formalin for 48 h. The formalin-fixed tissues were embedded in paraffin and then sliced into 4μm sections. Infiltrating neutrophils were stained using a Naphthol AS-D Chloroacetate Specific Esterase Kit (Sigma, St. Louis, MO, USA). We selected more than four fields of view at a primary magnification of ×200 in the cortex or hippocampus of every brain section. Cells were counted under a Nikon E100 microscope, and the data are presented as the means ± SEM.

### Intravital microscopy of the mouse brain

Intravital microscopy was performed as previously described [29]. Briefly, after anesthetization, the right parietal bone was subjected to craniotomy using a high-speed drill. Subsequently, the dura were removed from this site to expose the pial brain vessels. Rhodamine 6G (Sigma) was injected intravenously (0.5 mg/kg) into the mouse to label the leukocytes. Then, a microscope equipped with a fluorescent light source was used to detect the leukocytes. The data were collected through a sCMOS camera (ORCA-Flash 4.0, HAMAMATSU) mounted on the microscope and stored for subsequent analysis. Rolling leukocytes were defined as those cells moving at a slower velocity than the erythrocytes; adherent cells were defined as those that remained stationary for at least 30 s.

### RNA isolation and real-time quantitative PCR

After perfusion through the heart, the brains were homogenized in 1 ml of TRIzol (Takara Bio, Inc., Shiga, Japan) on ice, and RNA was extracted using TRIzol reagent according to the protocol supplied by the manufacturer. A total of 1 μg of total RNA was reverse-transcribed into cDNA. Then, SYBR® Green-based quantitative real-time polymerase chain reaction (PCR) was performed with a Bio-rad CFX 96 Touch (Bio-Rad Laboratories, Hercules, CA, USA) according to the manufacturer’s instructions. β_2_-Macroglobulin (β_2_-MG) was used as a housekeeping gene because its expression was not influenced by the treatments. The amplification conditions were as follows: 95 °C (2 min) followed by 32 cycles of 95 °C (20 s), 57.2 °C (30 s), and 72 °C (30 s). Quantitative PCR assays were conducted in triplicate for each sample and were performed using the 2^−ΔΔCt^ method. The amplified products were verified on a 1.5 % agarose gel by electrophoresis. The data are expressed as the *n*-fold differences relative to the standard.

### Western blotting

After i.c.v. LPS injection, the mice were anesthetized and perfused with ice-cold PBS to clear blood-borne proteins. Next, the brain was homogenized in 1 ml of cold PBS on ice, and the homogenate was centrifuged (12,000 rpm, 5 min). Cells were digested with radioimmunoprecipitation assay (RIPA) lysis buffer (50 mmol/L Tris–HCl, 150 mmol/L NaCl, 1 % Nonidet-40, 0.5 % sodium deoxycholate, 1 mmol/L EDTA, 1 mmol/L PMSF) for 30 min on ice and centrifuged at 12,000 rpm for 15 min at 4 °C. The brain homogenates or cell lysate were diluted in PBS and loading buffer, boiled (100 °C, 10 min), loaded on a 10 % acrylamide–SDS gel, and transferred to a Protran nitrocellulose membrane (Millipore, Billerica, MA, USA). The membranes were blocked with 5 % dry milk in PBS for 2 h at room temperature, incubated in primary antibodies against P-selectin (ab178424, Abcam, Cambridge, USA), E-selectin (ab18981, Abcam),VCAM-1 (ab134047, Abcam), ICAM-1 (ab25375, Abcam), CXCR2 (ab14935, Abcam), albumin (ab19194, Abcam), and β-actin (Cell Signaling, Beverly, CA, USA) overnight at 4 °C, washed, incubated in species-appropriate HRP-conjugated secondary antibodies for 1–2 h at room temperature in the dark, and washed three times. Then, the membranes were subjected to immunodetection using enhanced chemiluminescence reagents (PerkinElmer, Waltham, MA, USA).

### Determination of albumin concentrations in brain parenchyma

The mice were anesthetized and perfused with 20 ml of cold PBS over a period of 10 min to remove proteins from the blood circulation. Then, the concentration of albumin, a serum protein that is normally excluded from the brain by the intact blood–brain barrier, was measured in brain homogenates by Western blotting as previously described [30].

### Primary culture of purified microglia and astrocytes

After the neonatal cerebra were harvested, cerebral cortices devoid of cerebella, white matter, and leptomeninges were trypsinized for 5 min and then filtered through a 70-μm pore size filter. The cells from seven cerebra were seeded on an uncoated 75-cm^2^ culture flask and incubated in 40 ml of Dulbecco’s modified essential media (DMEM)/F12 containing 10 % FBS. The medium was replenished every 3–4 days after cell seeding. On days 13–14, microglia were isolated by shaking the flask at 250 rpm for 1 h as described [31]. Then, the cells were centrifuged and seeded at the appropriate density in six-well plates for further stimulation. After the mixed glial cells were passaged two to three times and shaken at 250 rpm for 6 h, the supernatants were discarded; the remaining adherent cells that remained consisted predominantly of astrocytes [32].

### Isolation and culture of primary mouse brain microvascular endothelial cells

Primary cerebral endothelial cells were prepared as previously described [33]. In brief, cortices from 7- to 8-week-old C57BL/6J mice were isolated by removing the cerebellum, striatum, optic nerves, and white matter. The outer vessels and the meninges were removed using dry cotton swabs. Then, the tissue sample was fragmented into 2-mm^2^-thick pieces and digested in 15 ml of 0.1 % collagen B (Roche, Indianapolis, IN, USA) supplemented with 30 U/ml DNase I (Sigma, St. Louis, MO, USA) for 1.5–2 h at 37 °C with occasional agitation. The suspension was centrifuged at 1000 rpm for 8 min. The resulting homogenate was mixed with 20 % BSA in DMEM and centrifuged at 4000 rpm for 20 min at 4 °C. The neural component and the BSA layer were discarded, and the pellet containing the vascular component was further digested in 0.1 % collagenase/dispase (Roche, Indianapolis, IN, USA) supplemented with 20 U/ml DNase I for 1.5–2 h at 37 °C. The final microvessel pellets were resuspended in DMEM supplemented with 30 % FBS (Life Technologies, Carlsbad, CA, USA), 3 ng/ml bFGF (Peprotech, Rocky Hill, NJ, USA), 10 U/ml heparin, 100 U/ml penicillin, and 100 mg/ml streptomycin. The medium was refreshed every 2 days. The endothelial cells grew to confluency after 7 days. The purity of the endothelial cells was >93 %.

### Generation of chimeric mice

Prior to irradiation, the mice were treated with antibiotics with the intention of eliminating *Pseudomonas aeruginosa* from the gastrointestinal tract. Neomycin was added to the drinking water 2 weeks post-irradiation. The recipient mice were lethally irradiated with two doses of 500 rad (separated by 2–3 h) as previously described [34]. Bone marrow cells were harvested from both the femora and tibiae of the donor mice, and approximately 5–6 million cells were intravenously injected into the recipient mice. Bone marrow transfers were performed as follows: (1) bone marrow cells from the CXCR2^−/−^ mice were transferred into the wild-type (WT) mice (chimeric, expressing CXCR2 on only the non-hematopoietic cells) and (2) bone marrow cells from the WT mice were transferred into the CXCR2^−/−^ mice (chimeric, expressing CXCR2 on only the hematopoietic cells). All chimeric mice were used for intravital microscopy experiments 6–8 weeks after bone marrow transfer.

### CXCR2 blockade

To block endothelial CXCR2, WT mice were intravenously injected with CXCR2 antagonist SB225002 (Cayman, Ann Arbor, MI, USA) at a dose of 1 mg/kg 0.5 h prior to i.c.v. LPS injection [35]. The mice in the control group were intravenously injected with 1 % DMSO 0.5 h prior to i.c.v. LPS injection. SB225002 was dissolved in DMSO and diluted with 0.9 % saline, achieving a final concentration of 1 % DMSO.

### Statistical analysis

The data were analyzed using SPSS software (17.0 for Windows, IBM Inc., Chicago, IL, USA). Data shown represent the means ± standard error of the mean (SEM). Statistical significance was determined using Student’s *t* tests for comparisons between two groups or by one-way ANOVA with Bonferroni correction for multiple groups of treatments. The differences were considered to be significant when *P* < 0.05.

## Results

### Generation of TALEN-mediated CXCL1 knockout mice

**Fig. 1:**
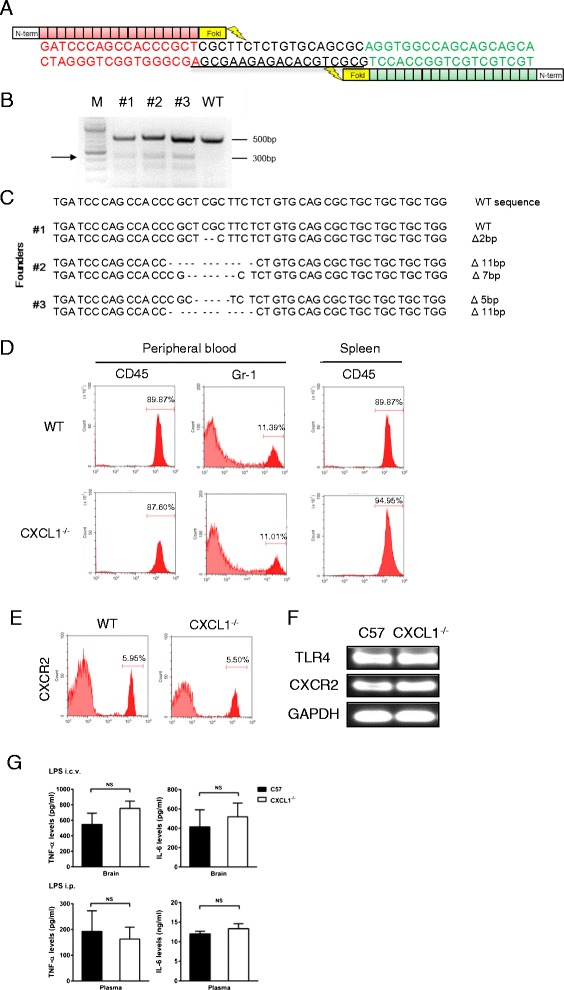
Generation of TALEN-mediated CXCL1 knockout mice. **A** DNA-binding sequences are presented in red or green, and the spacer region for CXCL1-TALEN where a double-strand break will occur is underlined. **B** T7 endonuclease I (T7EI) assays were conducted using genomic DNA from the founder mice. The arrow shows the size (300 bp) of T7EI-digested DNA fragments. #1, #2, and #3 are the mutant founder (F0) mice generated by injection with CXCL1-TALEN mRNA. **C** DNA sequences of the CXCL1 locus from live founder mice identified by T7E1 assays in B. “-” shows the deleted nucleotides. **D** Peripheral blood mononuclear cells and splenocytes were collected from wild-type or CXCL1^−/−^ mice. Numbers of CD45^+^ and Gr.1^+^ cells were quantified via flow cytometry. **E** Peripheral blood mononuclear cells were collected from wild-type or CXCL1^−/−^ mice, CXCR2 expression in neutrophils from WT and CXCR2^−/−^ mice were analyzed via flow cytometry. **F** mRNA expression levels of TLR4 and CXCR2 in the brain of WT or CXCL1^−/−^ mice were analyzed by RT-PCR. **G** Wild-type and CXCL1^−/−^ mice were treated with i.p. or i.c.v. LPS injection. Four hours later, levels of TNF-α and IL-6 in the brain tissue or plasma were determined by ELISA. Data are expressed as the mean ± SEM, *n =* 4 mice in all of the groups

To target the CXCL1 gene in the mouse genome, TALEN constructs that targeted the DNA sequence of the murine CXCL1 gene were created as illustrated in Fig. 1A. The founders (#1, #2, and #3) from the newborns were verified by T7 endonuclease I (Fig. 1B). All TALEN-induced mutations were deletions of variable lengths that induced frameshifts in the CXCL1 gene. Bi-allelic mutations were observed in three mutant mice (Fig. 1C). CXCL1^−/−^ mice were healthy, fertile, displayed no overt phenotype, and had normal leukocyte and neutrophil counts in the peripheral blood and spleen (Fig. 1D). Flow cytometry also revealed that neutrophils from CXCL1^−/−^ mice expressed CXCR2 at a similar level to wild-type mice (Fig. 1E). In addition, CXCL1^−/−^ mice showed normal TLR4 and CXCR2 mRNA expression levels in the brain (Fig. 1F). In response to systemic or i.c.v. LPS treatment, IL-6 and tumor necrosis factor-α (TNF-α) in the brain and plasma were increased to a similar extent in both CXCL1^−/−^ and wild-type mice (Fig. 1G).

### Intracerebroventricular injection of LPS induces neutrophil recruitment and CXCL chemokine (CXCL1, CXCL2, and CXCL5) expression

**Fig. 2:**
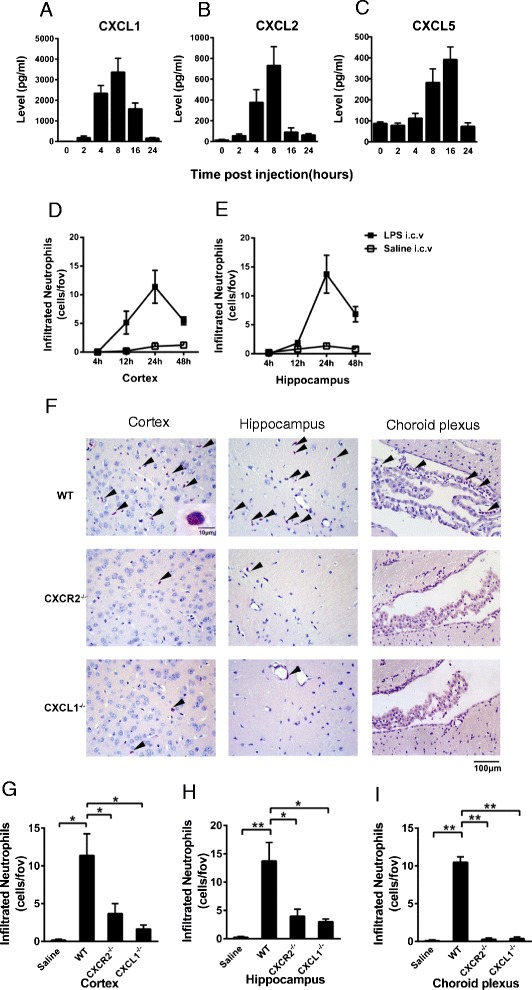
Chemokine levels and the effect of CXCR2 or CXCL1 deficiency on neutrophil recruitment to the brain parenchyma after i.c.v. LPS injection. Wild-type mice were i.c.v. injected with LPS. CXCL1 (**A**), CXCL2 (**B**), and CXCL5 (**C**) concentrations in the brains of WT (C57BL/6J) mice were determined via ELISA at various time points after i.c.v. LPS injection. WT mice i.c.v. injected with saline for 24 h served as negative controls. Infiltrating neutrophils in the cortex (**D**) and hippocampus (**E**) were quantified via esterase staining of the brain sections 4, 12, 24, or 48 h after i.c.v. LPS or saline injection. (**F**) The WT, CXCR2^−/−^, and CXCL1 mice were i.c.v. injected with LPS 24 h before the quantification of infiltrating neutrophils. Representative photomicrographs of brain sections stained for esterase positive neutrophils (arrows) from wild-type, (**G–I**) CXCR2^−/−^ and CXCL1 mice. Scale bar: 100 μm; 10 μm (inset). CXCR2 and CXCL1 deficiency significantly reduced neutrophil recruitment in the cortex (**G**), hippocampus (**H**), and choroid plexus (**I**). The results are presented as the means ± SEM and represent a minimum of five mice per group. *P < 0.05; **P < 0.01

Intracerebroventricular injection of LPS strongly induced the expression of inflammatory cytokines and chemokines in the CNS. As shown in Fig. 2A–C, LPS injection significantly induced the expression of CXCL chemokines, including CXCL1, CXCL2, and CXCL5. The levels of both CXCL1 (Fig. 2A) and CXCL2 (Fig. 2B) gradually increased and peaked 8 h after LPS injection, then gradually decreased thereafter. The level of CXCL5 (Fig. 2C) peaked at 16 h. Among all of these CXCL chemokines, CXCL1 exhibited the highest expression level following LPS injection. The peak concentration of CXCL5 was 10-fold less than that of CXCL1. At 12 h after i.c.v. LPS injection, neutrophils began to migrate into the brain. Infiltrating neutrophils, as detected by esterase-specific staining, were observed 12 h post-treatment in the cortex (Fig. 2D); this infiltration peaked at 24 h in the cortex (Fig. 2D) and the hippocampus (Fig. 2E) and decreased thereafter.

### Deficiency in CXCR2 or CXCL1 affects neutrophil infiltration induced by the i.c.v. injection of LPS

To investigate the role of CXCR2 in neutrophil recruitment to the brain, we compared neutrophil infiltration in the WT and CXCR2^−/−^ mice after i.c.v. LPS injection (Fig. 2F). CXCR2^−/−^ mice displayed significantly reduced neutrophil infiltration into the cerebral cortical (Fig. 2G), hippocampal regions (Fig. 2H) and choroid plexus (Fig. 2I). As CXCL1 was most highly expressed during LPS-induced CNS inflammation, we generated CXCL1-deficient mice using the TALEN knockout technique and compared neutrophil infiltration in these mice with that in the WT mice. Interestingly, the CXCL1^−/−^ mice also exhibited a complete lack of neutrophil recruitment to the cortex, the hippocampus, and choroid plexus (Fig. 2G–I). Therefore, both CXCL1 and CXCR2 play essential roles in LPS-induced neutrophil recruitment into the brain.

### Deficiency in CXCR2 or CXCL1 affects leukocyte recruitment in brain vessels

**Fig. 3:**
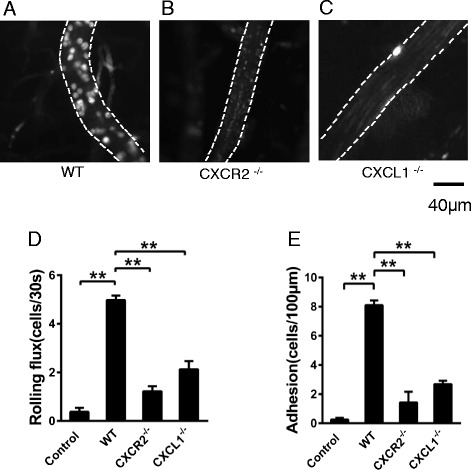
CXCR2 deficiency causes decreased leukocyte rolling and adhesion in brain vessels after i.c.v. LPS injection. Intravital microscopy was performed on wild-type (**A**), CXCR2^−/−^ (**B**), and CXCL1^−/−^ mice (**C**) 4 h after LPS i.c.v. injection. The results of rolling flux (**D**) and leukocyte adhesion (**E**) are presented as the means ± SEM. *n* = 4–6 mice per group. **P < 0.01

Chemokines regulate immune cell trafficking by assisting the activation, adhesion, crawling, and transmigration of leukocytes across the cerebral endothelial barrier. To verify the role of CXCR2 in the neutrophil recruitment cascade in brain microvessels, we performed intravital microscopy to examine leukocyte recruitment in brain vessels during LPS-induced CNS inflammation. As expected, i.c.v. LPS injection caused significant rolling and adhesion of leukocytes in post-capillary venules in brains of WT mice (Fig. 3A). Interestingly, leukocyte–endothelial cell interactions appeared to be reduced in the CXCR2^−/−^ (Fig. 3B) and CXCL1^−/−^ mice (Fig. 3C). Rolling (Fig. 3D) and adherent cells (Fig. 3e) were almost completely absent in the CXCR2^−/−^and CXCL1^−/−^ mice, indicating that both CXCL1 and CXCR2 are essential for the leukocyte recruitment cascade in cerebral microvessels in the LPS-induced neuroinflammation.

### CXCR2 deficiency decreases brain endothelial activation in vivo

**Fig. 4:**
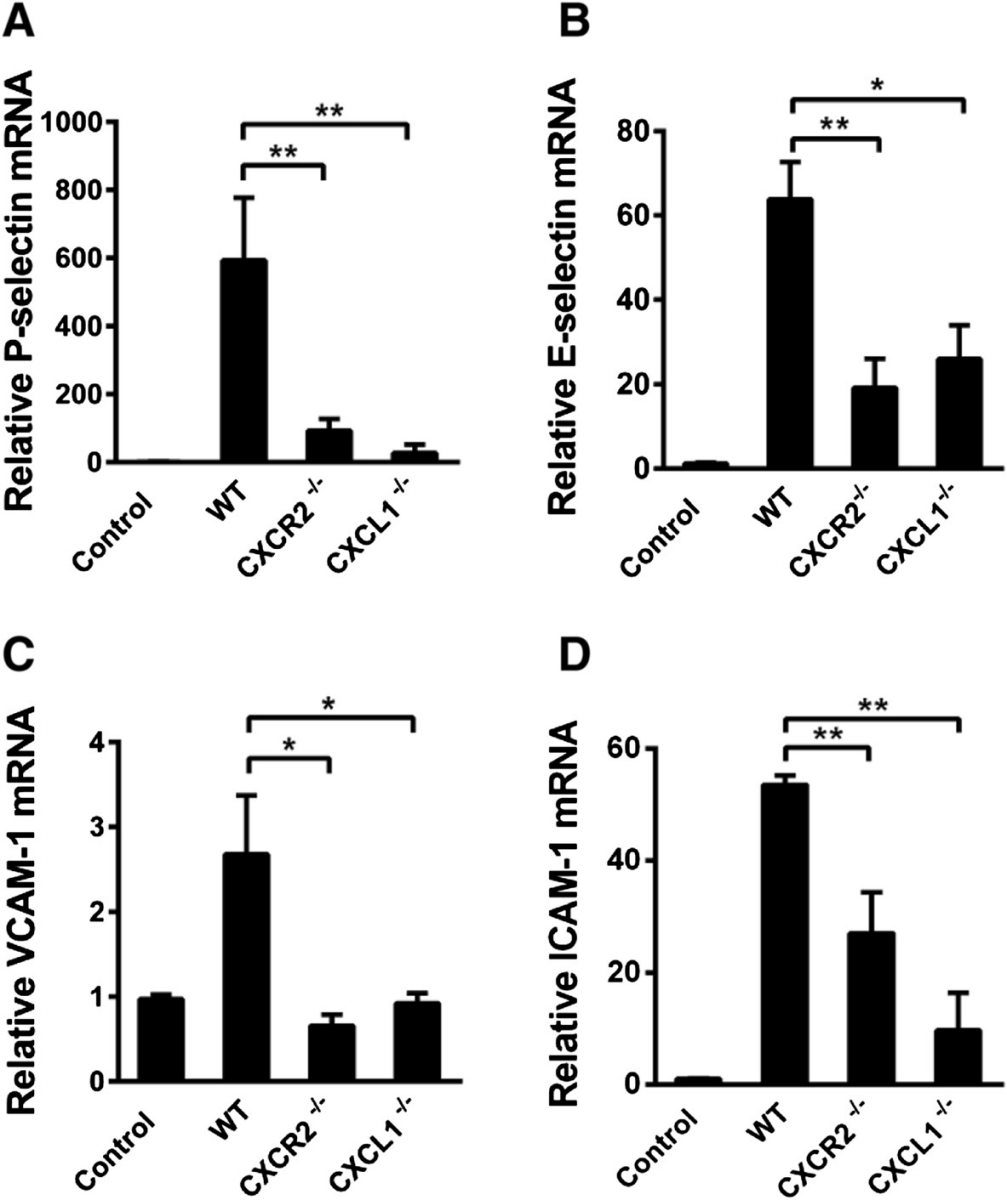
The effect of CXCR2 or CXCL1 deficiency on the mRNA expression of adhesion molecules in vivo. The mRNA expression of P-selectin, E-selectin, VCAM-1, and ICAM-1 saline-treated (4 h after i.c.v. saline injection) control group of WT mice and LPS-treated (4 h after i.c.v. LPS injection) WT, CXCR2^−/−^, and CXCL1^−/−^ mice was quantified via real-time PCR. Both CXCR2 and CXCL1 deficiency resulted in the down-regulation of P-selectin (**A**), E-selectin (**B**), VCAM-1 (**C**), and ICAM-1 (**D**) mRNA expression in the brain. *n* = 6–8 mice for all groups. *P < 0.05; **P < 0.01

**Fig. 5:**
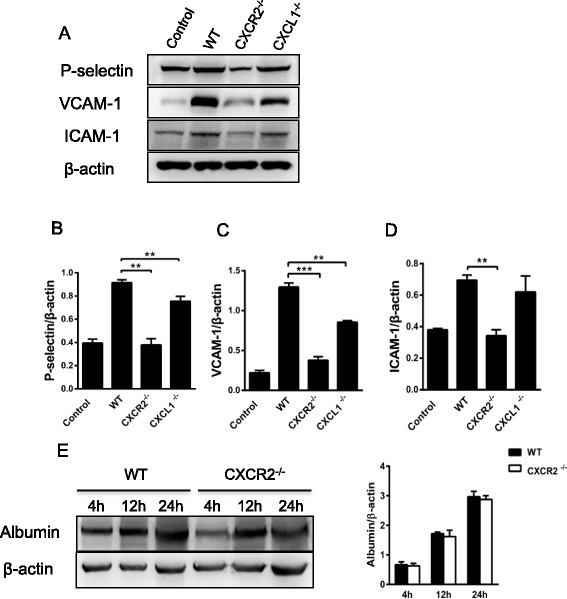
Effects of CXCR2 or CXCL1 deficiency on the expression of P-selectin, E-selectin, and adhesion molecules in vivo and on BBB permeability. **a** The protein expression of P-selectin, E-selectin, VCAM-1, and ICAM-1 (4 h after i.c.v. saline injection) in the saline-treated control group of WT mice and LPS-treated (4 h after i.c.v. LPS injection) WT, CXCR2^−/−^, and CXCL1^−/−^ mice was determined via Western blot analysis. Effects of CXCR2 or CXCL1 deficiency on P-selectin (**b**), VCAM-1 (**c**), and ICAM-1 (**d**) expression in the brain. **e** Western blotting analysis of the albumin levels in the brains of WT and CXCR2^−/−^ mice 4, 12, and 24 h after the intraventricular injection of LPS was performed. Optical densities were determined using a computer imaging analysis system. *n* = 5 mice per group. **P < 0.01; ***P < 0.001

To investigate the molecular mechanisms underlying the effects of CXCR2 and CXCL1 deficiency on leukocyte–endothelial cell interactions, the expression of adhesion molecules in CXCR2^−/−^ and CXCL1^−/−^ mice was assessed via real-time PCR 4 h after i.c.v. LPS injection. Interestingly, the mRNA expression levels of P-selectin (Fig. 4A), E-selectin (Fig. 4B), VCAM-1 (Fig. 4C), and ICAM-1 (Fig. 4D) were significantly reduced in CXCR2^−/−^ and CXCL1^−/−^ mice. Additionally, the protein expression levels of these adhesion molecules were assessed by Western blotting (Fig. 5A). Consistent with the real-time PCR results, both CXCR2 deficiency and CXCL1 deficiency dramatically reduced the protein expression of P-selectin (Fig. 5B) and VCAM-1 (Fig. 5C) in the brain. A significant reduction in the levels of ICAM-1 (Fig. 5D) was also observed in the CXCR2^−/−^ mice, albeit to a lesser extent. Taken together, these results suggest that both CXCR2 and CXCL1 are critical effectors that mediate the expression of adhesion molecules on cerebral endothelial cells during CNS inflammation.

It is well established that CNS inflammation induces permeability changes to the blood–brain barrier. The i.c.v. injection of LPS induced a significant change in brain albumin concentration 12 and 24 h post-treatment in both WT and CXCR2^−/−^ mice. However, no significant difference in albumin concentrations between the WT and CXCR2^−/−^ mice was observed 4, 12, and 24 h after i.c.v. LPS injection (Fig. 5E). Therefore, it is likely that rather than affecting the integrity of blood–brain barrier, CXCR2 deficiency affected neutrophil recruitment by attenuating endothelial activation.

### CXCR2 expression on CNS-residing cells mediates endothelial activation and leukocyte recruitment in chimeric mice

**Fig. 6:**
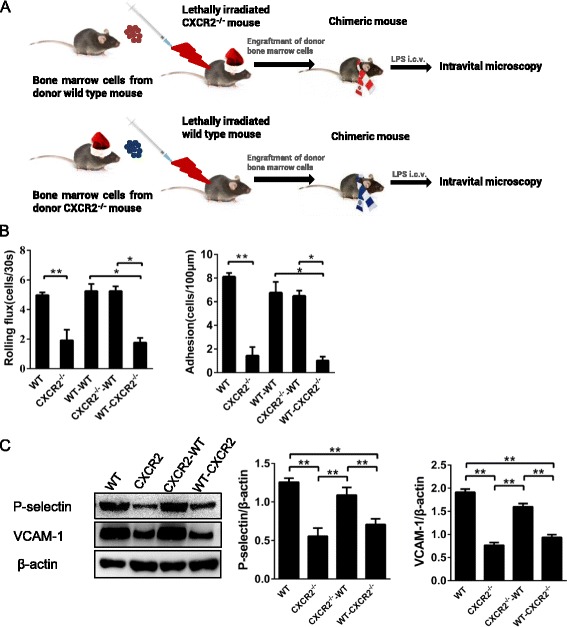
Leukocyte recruitment and the expression of P-selectin and VCAM-1 in the WT, CXCR2^−/−^, and chimeric mice. **a** Chimeric mice were generated by transferring bone marrow cells between WT and CXCR2^−/−^ mice. **b** Intravital microscopy was performed on WT, CXCR2^−/−^, and chimeric mice, 4 h after i.c.v. LPS injection. The number of rolling and adherent leukocytes is presented as the mean ± SEM. **c** The expression of P-selectin and VCAM-1 in the WT, CXCR2^−/−^, and chimeric mice was compared by Western blotting, *n* = 4 mice per group. Data are presented as the means ± SEM, *P < 0.05; **P < 0.01

To identify the source of functional CXCR2 that mediates leukocyte recruitment, we generated chimeric mice by transferring bone marrow cells between WT and CXCR2^−/−^ mice (Fig. 6A). Intravital microscopy was performed on all chimeric mice 4 h after i.c.v. LPS injection. CXCR2^−/−^ mice that were reconstituted using WT bone marrow cells exhibited reduced leukocyte rolling and adhesion. By contrast, the chimeric mice generated by reconstituting the WT mice using CXCR2^−/−^ bone marrow cells exhibited normal leukocyte recruitment to the cerebral microvessels (Fig. 6B). Consistent with our observations from intravital microscopy, the CXCR2^−/−^ mice reconstituted with WT bone marrow cells displayed significantly reduced levels of P-selectin and VCAM-1 expression, whereas the levels of P-selectin and VCAM-1 expression in the WT mice reconstituted using CXCR2^−/−^ bone marrow cells were almost similar to that of WT mice (Fig. 6C).

### Astrocyte-derived CXCL1 and endothelial CXCR2 are important in cerebral endothelial activation

**Fig. 7:**
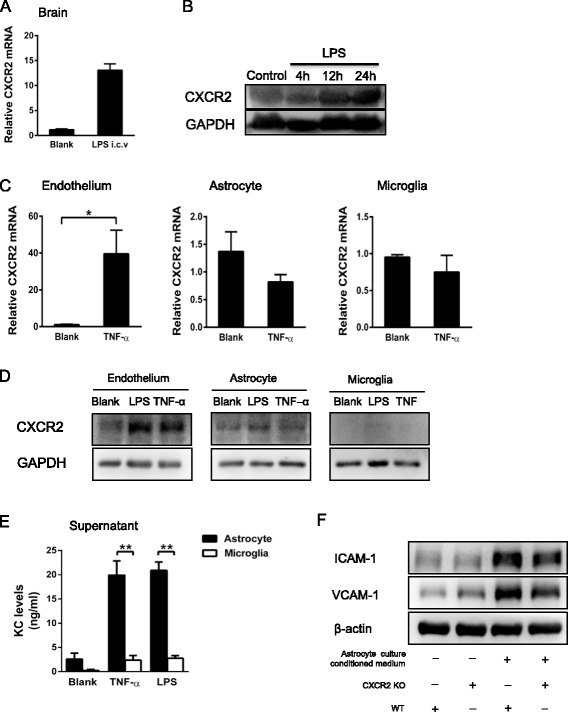
Astrocyte-derived CXCL1 and endothelial CXCR2 are essential for cerebral endothelial activation. **A** i.c.v. LPS injection (4 h) induced significant CXCR2 mRNA expression in WT mice. **B** Levels of CXCR2 protein after i.c.v. LPS injection from 4 to 24 h gradually increased compared with the control group (4 h after i.c.v. saline injection). **C** The expression of CXCR2 mRNA in primary brain microvascular endothelial cells, microglia, and astrocytes stimulated with either vehicle or TNF-α (100 ng/ml) was measured via real-time PCR. The results are represented as the means ± SEM of three independent experiments; *P < 0.05. **D** Primary endothelial cells, astrocytes, and microglia were seeded at 2 × 10^6^ cells/well in six-well plates and were incubated overnight. The following day, the cells were stimulated with 100 ng/ml LPS or 100 ng/ml TNF-α for 12 h. Cell lysates were collected and analyzed for CXCR2 expression via Western blotting. **E** Primary astrocytes and microglia from wild-type mice were seeded at 2 × 10^6^ cells/well in six-well plates and were incubated overnight. The following day, the cells were stimulated with 100 ng/ml LPS or 100 ng/ml TNF-α for 12 h. Then, the conditioned supernatants and cell lysates were collected and analyzed for CXCL1 expression via ELISA. The results are represented as the means ± SEM of three independent experiments; **P < 0.01. **F** Astrocyte culture conditioned medium was added into primary cerebral endothelial cells from wild-type or CXCR2^−/−^ mice, and the levels of VCAM-1 and ICAM-1 were measured via Western blotting

The reductions in endothelial activation and subsequent leukocyte–endothelial cell interactions in CNS microvessels resulted from a lack of CXCR2 expression on CNS-residing cells, but not on circulating neutrophils. Therefore, the functional CXCR2 that mediates endothelial activation is likely localized to radiation-resistant non-hematopoietic cells, such as endothelial or glial cells. LPS i.c.v. injection induced significant levels of CXCR2 mRNA (Fig. 7A) and protein (Fig. 7B). Upon TNF-α stimulation, primary endothelial cells, compared with glial cells, exhibited much higher expression of CXCR2 mRNA. No significant change in CXCR2 transcription was noted in primary microglia or astrocytes (Fig. 7C). Following stimulation with TNF-α or LPS, the cerebral endothelial cells, compared with astrocytes and microglia, also expressed much higher level of CXCR2 protein (Fig. 7D). Astrocytes secreted much higher levels of CXCL1 than microglia in response to TNF-α and LPS (Fig. 7E). Astrocyte culture conditioned medium stimulated strong expression of VCAM-1 and ICAM-1 in WT cerebral endothelial cells. Reduced expression of these molecules was observed in CXCR2^−/−^ endothelial cells (Fig. 7F). These results suggest that astrocyte-derived CXCL1 and endothelial CXCR2 do play critical roles in cerebral endothelial activation.

### Effect of CXCR2 blockade on leukocyte recruitment and endothelial activation

**Fig. 8:**
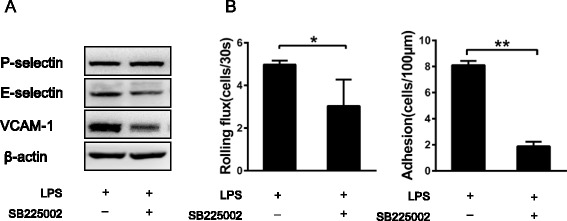
The effect of CXCR2 antagonist infusion on leukocyte rolling and adhesion in CNS vessels. WT mice received an intravenous injection of the CXCR2 antagonist SB225002 (1 mg/kg) 0.5 h prior to i.c.v. LPS injection. Four hours after i.c.v. LPS injection, the protein expression of P-selectin, VCAM-1, and E-selectin in the brain was determined by Western blot analysis (**A**). Intravital microscopy was performed on the mice. The results of leukocyte recruitment (**B**) are presented as the mean ± SEM. *n* = 4 mice for all groups. *P < 0.05; **P < 0.01

To validate the critical role of endothelial CXCR2 in cerebral endothelial activation, we intravenously infused the CXCR2 antagonist SB225002 at a dose of 1 mg/kg 0.5 h prior to i.c.v. LPS injection to block CXCR2 signaling [35] from the luminal surface of the cerebral microvessels. As detected by Western blotting, SB225002 treatment decreased the levels of VCAM-1 and E-selectin expression but not that of P-selectin (Fig. 8A). In addition, intravital microscopy also revealed that the injection of SB225002 significantly decreased leukocyte rolling and adhesion in brain microvessels (Fig. 8B). These results further indicate that endothelial CXCR2 plays a critical role in endothelial activation and subsequent leukocyte recruitment.

## Discussion

Leukocyte recruitment is a hallmark of various CNS inflammatory diseases. The chemokine receptor CXCR2 and its ligands CXCL1, CXCL2, and CXCL5 play crucial roles in the trafficking of neutrophils. In the current study, we showed that LPS injection into the brain significantly induced the production of CXCL1, CXCL2, and CXCL5. CXCL1, the most potent neutrophil-chemoattracting CXCR2 ligand, was upregulated in the CNS at the earliest time point and was correspondingly expressed at the highest level. The i.c.v. injection of LPS has been widely applied as an animal model for the study of brain inflammation. The dosage of 2 μg of LPS is considerably above what is observed in most infections and results in robust neutrophil recruitment. However, a significant reduction in the number of infiltrating neutrophils was observed in the brain parenchyma of CXCR2^−/−^ and CXCL1^−/−^ mice after i.c.v. LPS injection. Therefore, CXCL1 acts as the principal mediator of neutrophil recruitment during LPS-induced CNS inflammation.

The leukocyte recruitment cascade in brain vessels is directed by the complex interactions between adhesion molecules and their receptors [36, 37]. Intravital microscopy revealed that a deficiency in either CXCR2 or CXCL1 significantly reduced leukocyte–endothelial cell interactions in brain vessels. Additionally, reduced expression of P-selectin, VCAM-1, and ICAM-1 was observed in the brain of CXCR2^−/−^ mice. Therefore, it is likely that the functional CXCR2 that mediates leukocyte recruitment is located on the CNS endothelium. Our previous study reported that TNF-α in the LPS-treated brain activated the endothelium to cause an increase in adhesion molecule expression and leukocyte recruitment [29]. In response to i.c.v. LPS injection, CXCR2 deficiency did not reduce TNF-α levels in the brain. Clearly, a deficiency in CXCR2 or CXCL1 directly affected cerebral endothelial activation, but not microglial activation. In addition to its chemotactic properties, CXCL1 also exerts direct effects on BBB permeability. The exposure of brain microvascular endothelial cells to CXCL1 in vitro altered endothelial permeability and facilitated transendothelial monocyte migration [38]. However, in our study, CXCR2 deficiency did not affect albumin leakage across the BBB. Therefore, the reduction in neutrophil infiltration was not due to a change in the integrity of BBB but to a lack of cerebral endothelial activation resulting from CXCL1 or CXCR2 deficiency.

Among the chimeric mice generated by transferring bone marrow precursors between CXCR2^−/−^ and WT mice, WT mice reconstituted using CXCR2^−/−^ bone marrow cells exhibited normal cell recruitment to the brain vessels. Interestingly, functional CXCR2 is not expressed on circulating leukocytes from the bone marrow. Therefore, the activation of CXCR2 on leukocytes is not required for their recruitment in cerebral blood vessels. Earlier studies have identified the expression of CXCR2 on many types of CNS-residing cells [39–41]. This finding demonstrated that the expression of CXCR2 on CNS-residing cells, including endothelial cells, astrocytes, and microglia, is more important than its expression on circulating cells during CNS inflammation. Moreover, TNF-α robustly induced a high expression of CXCR2 mRNA in primary murine endothelial cells, but not in primary microglia or astrocytes. High levels of expression of the CXCR2 mRNA and protein were detected in wild-type cerebral endothelial cells, which strongly indicates that endothelial CXCR2 is a key player mediating cerebral endothelial activation. To further validate the role of endothelial CXCR2, we intravenously injected SB225002 to block the function of CXCR2 in brain endothelial cells, as SB225002 in the blood circulation can easily access the brain endothelium. Compared with mice treated with LPS alone, both cerebral endothelial activation and leukocyte recruitment in the cerebral vessels were reduced in the mice treated with both SB225002 and LPS. Taken together, these data indicate that SB225002 potently inhibited CXCR2 function on brain endothelial cells, thereby blocked leukocyte recruitment.

Astrocytes, which are more abundant than microglia in the brain [42], released much higher levels of CXCL1 than microglia in response to stimulation with LPS or TNF-α. Our study confirmed that astrocytes released significantly higher levels of CXCL1 than microglia in response to stimulation with LPS or TNF-α, suggesting that the main source of CXCL1 may be astrocytes. CXCL1 deficiency reduced leukocyte recruitment and endothelial activation by over 50 % in vivo. Astrocytes are essential structural components of the BBB [43, 44]; among all cell types in the brain, they have the easiest access to the brain endothelium and can release CXCL1, which possibly accumulate in the perivascular space at an extremely high concentration to activate cerebral endothelial cells. Therefore, the CXCL1 secreted from astrocytes and CXCR2 expressed on the endothelium may cooperate in contributing to cerebral endothelial activation and the subsequent leukocyte recruitment cascade during CNS inflammation.

## Conclusions

Endothelial activation is a critical step in the process of leukocyte recruitment during CNS inflammation. In the current study, we found that either CXCR2 or CXCL1 deficiency resulted in reduced neutrophil infiltration and leukocyte–endothelial cell interactions in the brain. A dramatic reduction in the endothelial expression of adhesion molecules was also noted in these mice. Our results demonstrate that CXCL1, an important factor secreted by astrocytes, also plays a critical role in leukocyte recruitment to the CNS by cooperating with CXCR2 expressed on cerebral endothelial cells. The CXCL1-CXCR2 axis may represent another potential therapeutic target for the treatment of CNS inflammatory diseases.

## Abbreviations

BBB: blood–brain barrier
CNS: central nervous system
CXCL: chemokine (CXC motif) ligand
CXCR2: CXC chemokine receptor 2
ELISA: enzyme-linked immunosorbent assay
i.c.v.: intracerebroventricular
IL: interleukin
LPS: lipopolysaccharide
PCR: polymerase chain reaction
TNF-α: tumor necrosis factor-α
WT: wild-type
β_2_-MG: β_2_-macroglobulin
